# 656. From Introduction to Integration: Evaluating the Physician Assistant Role in a Canadian Regional Burn Centre

**DOI:** 10.1093/jbcr/irag033.493

**Published:** 2026-04-06

**Authors:** Ashley Callahan, Kristine Laing, David Wallace, Alan D Rogers

**Affiliations:** Ross Tilley Burn Centre, Toronto, ON; Ross Tilley Burn Centre, Toronto, ON; Ross Tilley Burn Centre, Toronto, ON; Ross Tilley Burn Centre, Toronto, ON

## Abstract

**Introduction:**

Physician Assistants (PAs) are increasingly integrated into acute care teams, but despite evidence that Advanced Practice Providers improve efficiency, continuity, and outcomes in surgical and critical care settings, their role in specialized Canadian burn centres has not been formally evaluated. This study aimed to assess the impact, scope, and perceived value of a PA embedded within an American Burn Association–verified burn centre.

**Methods:**

An anonymous survey was distributed to all staff in a verified Canadian burn centre over a two-week period. The survey comprised Likert, frequency-based, and open-ended questions assessing scope awareness, clinical contributions, continuity, efficiency, and integration challenges. Quantitative results were summarized descriptively, and qualitative responses analyzed thematically.

**Results:**

Thirty-three staff responded, including nurses (61%), surgeons, intensivists, therapists, and allied health providers. Most reported at least daily PA interaction (82%). Scope awareness was inconsistent (36% full, 55% partial, 9% limited), with several respondents noting uncertainty regarding role boundaries and credentialing. Core contributions included line insertion (94%), admissions (91%), order writing (91%), progress notes (88%), discharge summaries (85%), and on-call coverage (85%). Respondents agreed that the PA improved continuity of care (72%), reduced physician burden (68%), enhanced multidisciplinary efficiency (65%), and expanded procedural access (61%). However, concerns included variable recognition of the PA role within the team, unclear authority in decision-making, limited opportunities for advanced training, and challenges in role standardization across shifts. Suggested improvements included clearer role delineation, formal education in advanced burn care (critical care, wound care certification, ABLS), structured integration into teaching and QI initiatives, and improved communication of scope to all staff. Overall satisfaction was high (82%), with strong support for ongoing employment (85%) and role expansion (52%).

**Conclusions:**

Integration of a PA into a Canadian burn centre was associated with high multidisciplinary satisfaction and broad recognition of improvements in continuity, efficiency, and procedural access. Nevertheless, variable scope awareness, inconsistent role recognition, and limited advanced training opportunities highlight areas for refinement. This represents the first structured evaluation of a PA in Canadian burn care and provides a framework for further development, education, and standardization of the role.

**Applicability of Research to Practice:**

This represents the first structured evaluation of a PA in Canadian burn care, providing a framework for further role development, education, and expansion within multidisciplinary burn teams.

**Funding for the study:**

N/A.

